# Alleviating Effects of Black Soybean Peptide on Oxidative Stress Injury Induced by Lead in PC12 Cells via Keap1/Nrf2/TXNIP Signaling Pathway

**DOI:** 10.3390/nu14153102

**Published:** 2022-07-28

**Authors:** Ning Li, Liuding Wen, Tiange Li, Huijie Yang, Mingwu Qiao, Tianlin Wang, Lianjun Song, Xianqing Huang, Mingming Li, Erkigul Bukyei, Fangyu Wang

**Affiliations:** 1 College of Food Science and Technology, Henan Agricultural University, 63#Agricultural Road, Zhengzhou 450000, China; liuding1215@126.com (L.W.); tiange@henau.edu.cn (T.L.); yanghj83862083@163.com (H.Y.); mingwu0309@163.com (M.Q.); wangtianlin@henau.edu.cn (T.W.); slj69@126.com (L.S.); hxq8210@126.com (X.H.); li2367494767@163.com (M.L.); 2 Department for Food Engineering and Hydromechanics, School of Engineering and Technology, Mongolian State University of Life Sciences, Zaisan-53, Ulaanbaatar 17024, Mongolia; erkigul@muls.edu.mn; 3 Key Laboratory for Animal Immunology, Henan Academy of Agricultural Sciences, 116#Huayuan Road, Zhengzhou 450002, China

**Keywords:** black soybean peptide, Pb, PC12 cells, Keap1/Nrf2/TXNIP, oxidative stress

## Abstract

Many researchers have found that Pb exposure can cause oxidative stress damage to the body’s tissue. Black soybean peptide (BSP) has a variety of physiological functions, especially in terms of oxidative stress. Nevertheless, the mitigation function of BSPs on Pb-induced oxidative stress damage in PC12 cells has not been clearly defined. In this study, cell viability was detected by CCK8. Oxidative stress indicators, such as ROS, GSH/GSSG, MDA, SOD, CAT, GPx, and GR, were tested with biochemical kit. Protein expression of Keap1, Nrf2, and TXNIP was measured by Western blot. Compared with the control group, Pb reduced the cell viability of PC12 cells. However, BSP treatment significantly increased the viability of PC12 cells induced by lead exposure (*p* < 0.05). Lead can enrich the contents of MDA and ROS, but decrease the amount of CAT, SOD, GR, GPx, and GSH/GSSG in PC12 cells, while BSP can alleviate it (*p* < 0.05). Lead can enhance the expression of Keap1 and TXNIP proteins, but reduce Nrf2 expression. In contrast, BSPs reversed this phenomenon (*p* < 0.05). BSPs can alleviate oxidative stress injury induced by lead in PC12 cells through the Keap1/Nrf2/TXNIP signaling pathway.

## 1. Introduction

Lead (plumbum (Pb)) has been shown to be a typical neurotoxic substance in many occupations [1]. Pb exposure causes neurotoxic effects, which can lead to various neurocognitive dysfunctions. In particular, occupational lead exposure in adults is associated with decreased cognitive abilities, including working memory. Pb is a known neurotoxic substance that may impair spatial learning and memory by damaging hippocampal long-term enhancement (LTP) and damage hippocampal neurons [2]. Pb exposure has been linked to high blood pressure, myocardial infarction, stroke, and arrhythmias in humans and animals [3]. It has been found that Pb exposure may cause cellular oxidative stress, ECG abnormalities, and elevated blood pressure, which may cause cardiovascular disease [4]. Oxidative stress is a disturbance between the production of reactive nitrogen (RNS) and reactive oxygen species (ROS) and antioxidant defenses, thus leading to neurodegenerative diseases [5]. Induced oxidative stress plays a very important role in the pathogenicity of heavy metal pollutants. Nowadays, reducing oxidative stress or scavenging free radicals through natural antioxidants has few adverse effects on health and is considered a good and important way to combat oxidative damage in the body.

According to research, black soybeans have high nutritional value, and prepared black soybean peptides are more easily digested and absorbed by the human body than black soybeans and have a certain health-care effect. Certain peptide components extracted from black soybean by-products have significant antioxidant and anticancer activity, are expected to alleviate oxidative stress damage, and can be a good candidate for functional foods or related drugs [6]. It has been shown that there is an effect of soy protein hydrolysate on intracellular antioxidant activity, demonstrating that soy peptides can effectively activate Nrf2/antioxidant reaction element (ARE)-mediated activity [7]. Black soybean protein has high water absorption and significant (*p* < 0.05) growth inhibition against ovarian cancer (SKOV3) and liver cancer cell lines (SMMC-7721). Black bean bark anthocyanin extract (BSSCE) has strong α-amylase inhibitory activity and inhibits the accumulation of denatured fat in HepG2 cells (liver cancer cells).

PC12 is a mature rat adrenal pheochromocytoma cell line that synthesizes and sets free dopamine [8]. One of the cell lines most used by researchers in neuroscience studies is the PC12 cell line [9]. Kudo TA has developed a new method to induce neuronal differentiation in rat PC12 cells by temperature-controlled repetitive thermal stimulation (TRTS) of heated plates [10]. It has been shown that oxidative stress induces iron sagging and mitochondrial dysfunction in PC12 cells [11]. It has been shown that hydroxytyrosol is effective on damage induced by oxidative stress in PC12 cells [12]. It has been shown that the neuroprotective effect of theaflavin on oxidative stress in PC12 cells stems from the inhibition of oxidase activity [13].

Keap1 (Kelch-like ECH-associated protein 1) is a protein sensor for oxidative stress [5]. It is a protein that inhibits the transcriptional activity of Nrf2 and is sensitive to electrophiles [14]. Nrf2 and its endogenous inhibitor Keap1 are a very common, relatively evolved intracellular defense mechanism that can be used to combat oxidative stress in cells [15]. The Keap1/Nrf2 pathway is the primary protective response to intracellular electrophilic and oxidative stress. Thioredoxin-interacting protein (TXNIP) is one of the α-arrestin proteins, known as the central regulator of glucose and lipid metabolism, which can participate in the regulation of related diseases, such as diabetes and inflammation. TXNIP is a negative regulator of thioredoxin (TRX) function and consequently favors oxidative stress [16]. Al-Mubarak et al. found that activating astrocyte Nrf2 through oxidative stress involves Keap1-independent nonnormative signaling, suggesting that activating Nrf2 through Keap1 inhibition of drugs may be an effective treatment [17]. The rational regulation of oxidative stress mediated by the Keap1/Nrf2 pathway will benefit a variety of illnesses associated with abnormal angiogenesis, such as hypertension and cancer [18]. Nrf2 activity was involved in oxidative stress response modulation via the Keap1/Nrf2/TXNIP signaling pathway [15,19].

**Figure 1:**
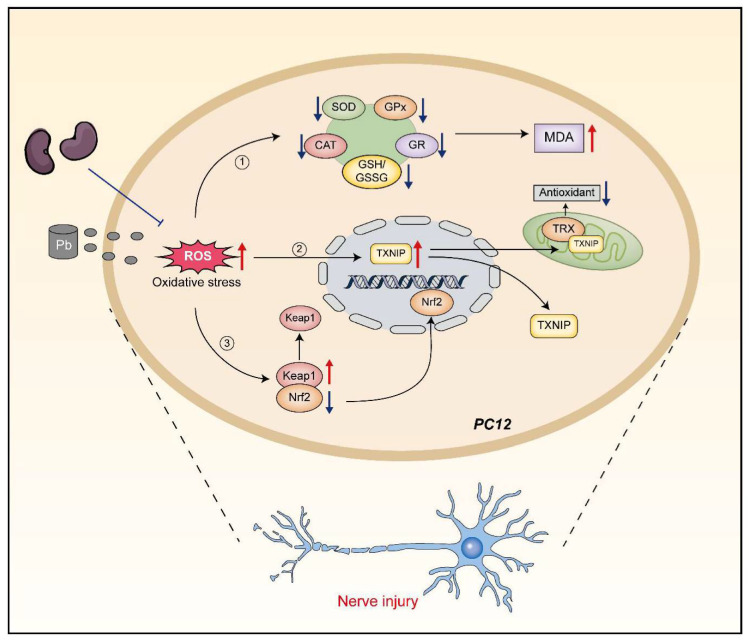
Mechanism of action of BSP1, BSP3, and BSP4 in mitigating oxidative stress due to lead exposure.

However, whether black soybean peptides alleviate Pb-induced oxidative stress response of PC12 cells and the role of Keap1, Nrf2 and, TXNIP proteins in it has not been reported. Therefore, this study investigated the mechanism of black soybean peptides in alleviating lead-induced oxidative stress and the role of the Keap1/Nrf2/TXNIP signaling pathway (Figure 1).

## 2. Materials and Methods

### 2.1. Materials

Lead acetate (PbC_4_H_6_O_4_·3H_2_O) and ascorbic acid were purchased from Aladdin Industrial Corporation (Shanghai, China). High-glucose Dulbecco’s modified Eagle’s medium (DMEM), fetal bovine serum (FBS), ROS assay kits, superoxide dismutase activity (SOD) assay kits, a GSH assay kit, and a micro-malondialdehyde (MDA) assay kit were bought from Solarbio (Beijing, China). The activities of CAT, GR, GPx, and GSSG were detected using the corresponding test kits (Beyotime Biotechnology, Nanjing, China). Primary antibodies against β-actin, Keap1, Nrf2, and TXNIP were purchased from Solarbio. Conjugated anti-rabbit and anti-mouse antibodies were bought from Proteintech (Wuhan, China). All other chemicals were of analytical grade and purchased from Solarbio and Beyotime unless otherwise stated.

The sequence information and references of the 5 BSPs are listed in Table 1. In this study, all peptides used were synthesized by GL Biochem (Shanghai, China), and their purity was greater than 80%. The bioactivity score of black soybean peptides was predicted by the online website PeptideRanker (http://distilldeep.ucd.ie/PeptideRanker/) (accessed on 15 December 2021), and a score greater than 0.5 indicated that they are likely to have high bioactivity. The net charge number and water solubility of black soybean peptides were determined by Innovagen (http://www.innovagen.com/proteomics-tools) (accessed on 15 December 2021) and DPL (http://www.peptide-ligand.cn/tools/) (accessed on 15 December 2021) online tools for prediction. When the value of net charge was ≥2 or ≤−2, it indicated that the black soybean peptide had good water solubility. The toxicity of black soybean peptides was predicted by ToxinPred (http://crdd.osdd.net/raghava//toxinpred/) (accessed on 15 December 2021).

### 2.2. Cell Culture and Treatment

Rat pheochromocytoma cell line PC12 cells were obtained from the Animal Immunology Laboratory, Henan Provincial Academy of Agricultural Sciences (Zhengzhou, China). Culture cells in DMEM and add 10% (*v*/*v*) heat-inactivated FBS to the culture medium in a 5% CO_2_ incubator at 37 °C. Logarithmic growth cells were reserved for follow-up experiments.

Lead acetate was dissolved in ultrapure water at a concentration of 10 mM. For use, the 10 mM lead solution was diluted with DMEM to the appropriate working concentration, and the cells were then incubated with the diluted lead solution. Black soy peptides and VC were treated in the same way.

PC12 cells at logarithmic growth stage were preincubated in 6-well plates for 24 h, then the medium was replaced with fresh medium with or without peptides for 4 h, and finally, the medium was replaced with lead for 24 h for subsequent assays. This experiment was divided into a control group, Pb group, Pb +BSP1 group, Pb +BSP3 group, Pb +BSP4 group, and VC group.

### 2.3. Cell Viability Assay

Cell viability was measured by Cell Counting Kit 8 (CCK8). First, PC12 cells in the logarithmic growth phase were incubated for 24 h at 10^4^ per well uniformly spread on a 96-well plate. Then, the medium in the wells was replaced with the corresponding concentration of BSPs/Pb and incubated for 4/24 h. Finally, 10 μL of CCK8 solution was added to each well and incubation was continued for 1 h. The absorbance at 450 nm was then measured.

### 2.4. Measurement of Antioxidant Enzymes

#### 2.4.1. CAT Activity Measurement

Incubate the cells to be measured in 2 mL serum-free RPMI 1640 medium and seed them in triplicate into a 6-well plate at 37 °C. Processing cells according to different groups. The cells are then lysed, and the protein concentration determined according to the Bradford method. Then, according to the instructions, measure CAT activity in cell lysates using the catalase assay kit [20].

#### 2.4.2. SOD Activity Measurement

This study used the Gianopulitis and Reice methods to determine the total activity of SOD, which gauged its capability to block the photochemical reduction of nitroblotriazole (NBT). The determination method of SOD enzyme activity is spectrophotometry. The test mixture for this experiment was 0.5 mL of reaction solution (50 mM phosphate buffer, EDTA 0.1 mM, 0.21 mM riboflavin, and 75 mM NBT) and 1 mL of PC cell fluid. The reaction cup containing the reaction solution is exposed to a fluorescent beam (20 watts of fluorescence) for 30 min. The absorbance of the sample is observed and recorded, and the blanks in the 560 nm wavelength range are controlled. Finally, the content of SOD is calculated according to the following formula: (OD test-OD Blank/OD Blank) × 100. Each SOD unit is the amount of enzyme required for 50% inhibitory photochemical recovery of NBT under experimental conditions [21].

#### 2.4.3. ROS Activity Measurement

Wash the cells to be measured, incubate the cells with 2.5 mM dihydroethidium (DHE, red) for 30 min at 37 °C, and stain the nuclei with Hoechst 33,342 (blue) for 30 min. The cells are then observed with a laser-scanning confocal microscope, and the images are analyzed with Image-Pro Plus software (version 6.0). Calculate the average fluorescence intensity of each cell, emit signals on average cells per field, and perform data analysis [22].

#### 2.4.4. MDA Activity Measurement

The PC12 cells were washed twice with PBS. Then, they were treated with Pb, peptide and Pb + different soybean peptides, respectively. Finally, collect cells by MDA detection kit and follow the kit instructions to detect MDA levels [7].

#### 2.4.5. Measurement of Glutathione (GSH) and Oxidized Glutathione (GSSG)

The PC12 cells were treated with Pb, peptide, and Pb + different soybean peptides, respectively. Then, the ratio of GSH/GSSG is determined with a luminometer kit according to the manufacturer’s instructions [11].

#### 2.4.6. GPx Activity Measurement

When the activity of GPx is measured, it converts the decreased GSH into GSSG. Nicotinamide adenine dinucleotide phosphoric acid (NADPH) is oxidized to NADP^+^ to obtain compounds, the absorbance of these compounds at 340 nm is measured, and the activity of GPx is determined by a decrease in absorbance. The absorbance of samples was measured five times every 5 min at 340 nm.

#### 2.4.7. GR Activity Measurement

The experiment was used to determine the activity of GR with a GR analysis kit. The principle of this kit is that NADPH can reduce the expression of GSSG at 340 nm when GR is present, measured 5 times every 1 min, and the results are expressed in nmol/min/mL [23].

### 2.5. Expression of Keap1/Nrf2/TXNIP Pathway-Related Proteins

Extract the nuclear and cytoplasmic proteins in the cells using the nuclear extraction kit by referring to the method provided by the manufacturer. And then use the BCA Protein Analysis Kit to determine the total protein concentration of these extracted proteins. Firstly, add the loading buffer to the protein sample and boil at 50 μg/well for 10 min at 95 °C. Secondly, use a 10% polyacrylamide gel to separate the protein, shift it to a polyvinylidene fluoride (PVDF) membrane, and store it in 5% bovine serum albumin (BSA) for 1 h at room temperature. Primary antibodies are diluted at 1:1800, and reacted overnight at 4 °C, containing β-actin proteins Keap1, Nrf2, and TXNIP. Then, wash the membrane 3 times with TBS, 0.05% Tween 20 (TBST) for 5 min, followed by incubation with the secondary antibody (1:1000 dilution, Abcam) for 1 h. Finally, rinse the membrane again 3 times × 5 min. The β-actin protein was used as an endogenous reference gene, the expression of the target protein was tested by the gel image processing system, and the image checked the gray-scale value of the target band. Every experiment was performed three times independently [24].

### 2.6. Statistical Analysis

All data results were independently measured at least three times. Data are shown as means ± standard deviation (SD) of the different independent experiments. Where it was necessary to compare three or more groups of data, LSD tests and one-way ANOVA can be used. When statistically analyzing data, the SPSS 20 statistical package can be used. When *p* < 0.05, the experimental results were considered significant.

## 3. Results and Discussion

### 3.1. Preventive Effects of BSPs on Cell Viability with Pb Exposure

Cell viability is generally considered the number of healthy cells in a sample. Typically, the same analysis used to detect cell viability over a certain period can reflect cell proliferation. For example, cell viability can link cell behavior to cell numbers by providing a trusted picture of anabolic activity [25].

**Figure 2:**
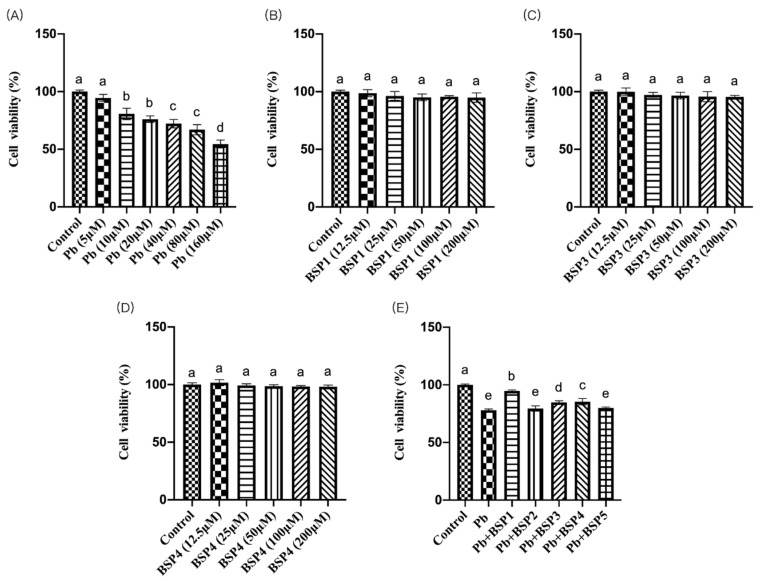
Cell viability assay. (**A**) Effects of lead on cell viability in PC12 cells. PC12 cells were incubated with lead (0–160 μM) for 24 h. (**B**–**D**) Effects of BSP1, BSP3, and BSP4 cell viability in PC12 cells. PC12 cells were incubated with (**B**) BSP1, (**C**) BSP3, and (**D**) BSP4 for 4 h. (**E**) Effects of BSP1–5 on cell viability in lead-exposed PC12 cells. PC12 cells were preincubated with BSP1–5 (200 μM) for 4 h, then treated with 10 μM lead for 24 h. The value of the bars indicate the means ± SD (*n* = at least 3). The same letter means there is no significant difference, and different letters mean there is a significant difference (*p* < 0.05).

This study found that the cell viability of PC12 cells in the Pb (10 μM) group was much lower than that in the control group, and the difference was statistically significant (*p *< 0.05) (Figure 2A). This concentration has also been used in several studies [26,27]. We selected a concentration of 10 μM lead for the subsequent detection. Then, PC12 cells were incubated with different concentrations of black soybean peptide for 4 h to observe toxicity. Figure 2B–D shows that 12.5–200 μM of black soybean peptide did not produce toxicity to PC12 cells (*p *< 0.05), so the maximum action concentration was chosen for the subsequent experiments. Black soybean peptides 1–5 were preincubated for 4 h and then incubated with lead for 24 h to screen the protective black soybean peptides. The cell viability of PC12 cells in BSP1, BSP3, and BSP4 black soybean peptide treatment groups was much higher than that in the lead exposure group, and the difference was also statistically significant (*p *< 0.05) (Figure 2E). Therefore, in this study, BSP1, BSP3, and BSP4 were selected for the follow-up assays.

In this study, we attempted to elucidate the effects of Pb exposure and black soybean peptides on cell viability and their interaction mechanisms. Our study revealed that Pb exposure greatly inhibited cell viability in PC12, while black soybean peptides can reduce the damage of lead exposure to cell viability. In the latest research on the effects of Pb accumulation on cell viability and apoptosis, it was shown that environmental exposure to low concentrations of Pb might greatly impair the function of macrophages, and an increase in the number of apoptotic cells may cause the occurrence of many diseases in the organism [28]. It has been found that when intracellular ROS increases, cell viability decreases, especially when the metal mixture is used as a promoter stimulus [29].

### 3.2. Preventive Effects of BSPs on ROS Generation

Reactive oxygen species (ROS) are after-products of the aerobic metabolism of species, which include free radicals, peroxides, and oxygen ions [30]. The pro-oxidation process’s essence is producing ROS and nitrogen species (RNS). Large amounts of ROS can cause DNA damage, lipid peroxidation, or protein alterations, which is a known factor in many diseases, such as cardiovascular disease and neurological disease [21]. As blood-lead levels rise, lead-induced cellular oxidative stress generates ROS, inducing autophagy and damaging endothelial cells [4].

**Figure 3:**
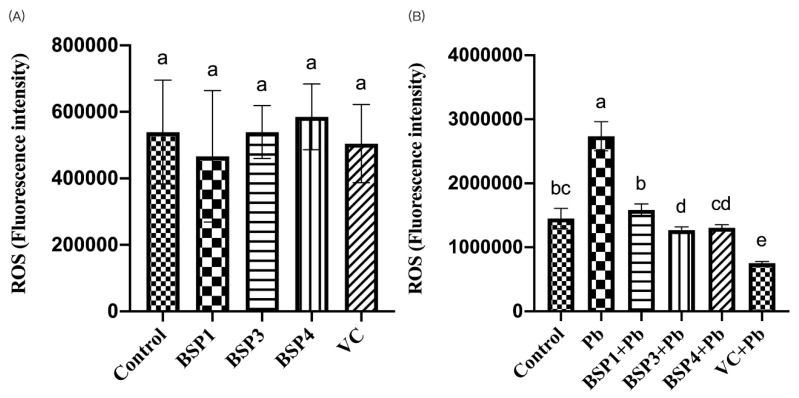
Effects of BSP1, BSP3, BSP4, and VC on intracellular ROS in PC12 cells. (**A**) PC12 cells were incubated with BSP1, BSP3, BSP4, and VC for 4 h. (**B**) PC12 cells were preincubated with BSP1, BSP3, BSP4, and VC for 4 h, then treated with lead for 24 h. The value of the bars indicate the means ± SD (*n* = at least 3). The same letter means there is no significant difference, and different letters mean there is a significant difference (*p* < 0.05).

Ascorbic acid (VC) has a powerful antioxidant effect. By reviewing previous studies, we chose 80 μM of VC as a positive control group to evaluate the antioxidant capacity of black soybean peptides [31]. In this study, black soybean peptides and VC were incubated separately in PC12 cells to observe their effect on ROS. BSP1, BSP3, BSP4, and VC did not significantly increase the level of ROS compared to the control group (*p* < 0.05) (Figure 3A). Then, black soybean peptides and VC were preincubated for 4 h before adding lead to observe their effects on ROS. Compared with the control group, the expression of ROS in the Pb group increased by 88%, and the difference was statistically significant (*p* < 0.05). However, the ROS content in these three black soybean peptides + Pb groups were reduced by 79%, 101%, and 98% compared with the Pb group, and the difference was statistically significant (*p* < 0.05). The ROS content in the VC group also showed an improvement of 130% compared with the Pb group (*p* < 0.05) (Figure 3B).

If a large amount of ROS is produced in an organism, it can lead to pathology in the organism. Therefore, we explored whether lead exposure in PC12 cells can induce ROS production and whether black soybean peptides can reduce cell damage and ROS production. The results showed that these three black soybean peptides we screened out could alleviate the abnormal production of Pb-induced ROS and restore cells to a certain extent. Guo et al. have shown that Pb^2+^-induced damage propagates through an intercellular junction communication (GJIC) between PC12 cells while inducing apoptosis in bystander cells through ROS mitochondrial-dependent apoptosis signals [23]. Yuan et al. said SOD and GSH/GSSG quickly cleared the ROS within cells to keep the stability of the internal structures of the cells and tissue. Otherwise, high ROS levels will disrupt the cell balance [28].

### 3.3. Effect of Antioxidant Enzymes and MDA by BSPs

SOD is the first line of defense against oxygen radicals and is one of the most important antioxidant enzymes. It can catalyze the breakdown of superoxide anions into H_2_O_2_. Hydrogen peroxide can be converted to O_2_ and H_2_O by CAT. Reactive oxygen species can damage macromolecules, including cellular proteins, nucleic acids, and membrane lipids [22]. SOD is an essential free-radical scavenger in the human body that protects brain tissue from free radical damage. At the same time, MDA is an oxygen-free radical-induced lipid peroxidation product that indirectly reflects the degree of cell damage [27]. As a biomarker of lipid peroxidation and one of the main products of lipid H_2_O_2_, MDA can lead to cross-linking and polymerization of active macromolecules, finally bringing about severe cytotoxicity [7].

**Figure 4:**
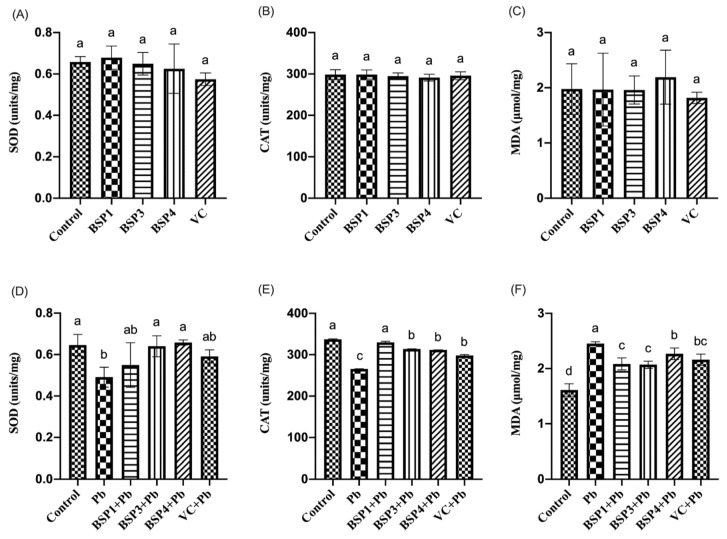
Effect of BSP1, BSP3, BSP4, and VC on SOD/CAT/MDA in PC12 cells. (**A**–**C**) PC12 cells were incubated with BSP1, BSP3, BSP4, and VC for 4 h. (**D**–**F**) PC12 cells were preincubated with BSP1, BSP3, BSP4, and VC for 4 h, then treated with lead for 24 h. SOD results are shown in (**A**,**D**). CAT results are shown in (**B**,**E**). MDA results are shown in (**C**,**F**). The value of the bars indicates the means ± SD (*n* = at least 3). The same letter means there is no significant difference, and different letters mean there is a significant difference (*p* < 0.05).

In this study, black soybean peptide and VC were incubated separately in PC12 cells to observe their effect on SOD, CAT, and MDA. Black soybean peptides (BSP1, BSP3, BSP4) and VC did not significantly change the level of SOD, CAT, and MDA compared to the control group (*p* < 0.05) (Figure 4A–C). Then, black soybean peptides and VC were preincubated for 4 h before adding lead to observe their effects on SOD, CAT, and MDA. This study found that the SOD expression in the Pb group was reduced by 34% compared with the control group, and the difference was statistically significant (*p* < 0.05). Compared with the Pb group, the expressions of SOD in the BSP3+Pb and BSP4+Pb groups were enhanced by 23% and 26%, and the difference was statistically significant (*p* < 0.05). However, although the SOD content in the BSP1+Pb group and VC group increased compared with lead group, the difference between them was not statistically significant (*p >* 0.05) (Figure 4D). Compared with the control group, the expression of CAT in PC12 cells was decreased by 32% in the Pb group, and the difference was statistically significant (*p* < 0.05). The CAT expression level in these three black soybean peptides + Pb group was higher by 19%, 15%, and 14% than in the Pb group, and the difference was statistically significant (*p* < 0.05). The CAT expression level in the VC group also showed an improvement of 10% compared with Pb group (*p* < 0.05) (Figure 4E). Compared with the control group, the expression of MDA in PC12 cells in the Pb group increased by 52%, and there was a statistical difference (*p* < 0.05). The MDA expression in these three black soybean peptide + Pb groups were reduced by 23%, 24%, and 12% compared with the Pb group, and there was a statistical difference (*p* < 0.05). The MDA expression level in the VC group also showed an improvement by 18% compared with Pb group (*p* < 0.05) (Figure 4F).

SOD is an important member of the antioxidant enzyme system in biological systems. CAT can oxidize some cytotoxic substances, inseparable from the occurrence and development of many diseases. As the end product of the peroxidation reaction, MDA’s content can indirectly reflect the degree of cell damage. In this experiment, Pb and black soybean peptides were used as influencing factors to explore changes in SOD, CAT, and MDA content in PC12 cells. The results showed that Pb exposure would damage cells, reduce the expression of SOD and CAT, and enhance the expression of MDA. At the same time, black soybean peptides alleviated the abnormal index changes induced by Pb, increased the production of SOD and CAT, and reduced the production of MDA. Recent studies have shown that after CAT and GPx work, SOD’s free-radical scavenging activity is effective, because its dismutase activity produces hydrogen peroxide from superoxide ions. It is much more toxic than oxygen-derived free radicals, requiring CAT and GPx to remove this. It has been found that BSSCE can promote glucose metabolism by regulating glycogen synthesis in diabetic mice, and SOD, CAT, and GPx activity in mouse livers was also greatly improved [32].

### 3.4. Effect of GPx, GR, and GSH/GSSG by BSPs

GPx is a selenium-containing enzyme whose biochemical function is to reduce lipid peroxides to corresponding alcohols and free H_2_O_2_ to water. The response is essential in protecting cells from free radical damage formed by hydrogen peroxide breakdown. GSH is an atypical tripeptide class that detoxifies exogenous substances because it acts as a nucleophilic co-matrix of glutathione-S-transferase. It is also a basic electron donor for GPx enzymes. GR is a flavin protein whose function is to catalyze NADPH-dependent reduction of GSSG to GSH, which is necessary to maintain adequate cellular GSH contents [23]. GSH-Px can catalyze glutathione from decreased GSH to oxidized GSSG, and GSH consumption and GSSG production reflect the degree of intracellular oxidation. If GSH is missing, it directly activates lipoxygenase and inhibits the activity of Gpx4, leading to lipid peroxidation. Gpx4 has been recognized as a significant regulator of iron sagging in some cancer cells [33].

**Figure 5:**
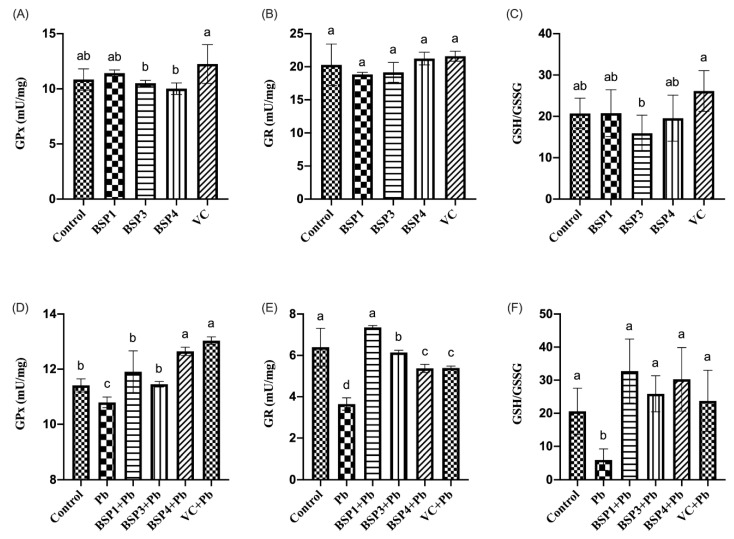
Effect of BSP1, BSP3, BSP4, and VC on GPx/GR/GSH/GSSG in PC12 cells. (**A**–**C**) PC12 cells were incubated with BSP1, BSP3, BSP4, and VC for 4 h. (**D**–**F**) PC12 cells were preincubated with BSP1, BSP3, BSP4, and VC for 4 h, then treated with lead for 24 h. The results of GPx are shown in (**A**,**D**). The results of GR are shown in (**B**,**E**). The results of GSH/GSSG are shown in (**C**,**F**). The value of the bars indicates the means ± SD (*n* = at least 3). The same letter means there is no significant difference, and different letters mean there is a significant difference (*p* < 0.05).

In this study, black soybean peptide and VC were incubated separately in PC12 cells to observe their effect on GPx, GR, and GSH/GSSG. Black soybean peptides (BSP1, BSP3, BSP4) and VC did not significantly change the level of GPx, GR, or GSH/GSSG compared to the control group (*p* < 0.05) (Figure 5A–C). Then, black soybean peptides and VC were preincubated for 4 h before adding lead to observe their effects on GPx, GR, and GSH/GSSG. GPx content in PC12 cells in the Pb group was reduced by 6% compared with that in the control group, and the difference was statistically significant (*p* < 0.05), but the contents of GPx in the BSP1+Pb, BSP3+Pb and BSP4+Pb groups were enhanced by 10%, 6%, and 16% compared with the lead-exposure group, especially in the BSP4+Pb group, and the difference was statistically significant (*p* < 0.05). Compared with the BSP1+Pb and BSP3+Pb groups, the expression level of GPx in the BSP4+Pb group was greatly enhanced, and the difference was statistically significant (*p* < 0.05). In contrast, compared with the VC group, there was no statistical difference (*p* > 0.05) (Figure 5D). GR expression in the Pb group was reduced by 43% compared with that in the control group (*p* < 0.05). GR content in BSP1+Pb, BSP3+Pb and BSP4+Pb groups was enhanced by 57%, 39%, and 26% compared with the lead exposure group, and the difference was statistically significant (*p* < 0.05). The content of GR in BSP1+Pb group was the highest, and there were no statistical differences compared to the control group (*p* > 0.05). The GR expression level in VC group also showed an improvement of 25% compared with Pb group (Figure 5E) (*p* < 0.05). The content of GSH/GSSG in the Pb group was reduced by 71% compared with that in the control group, and the difference was statistically significant (*p* < 0.05). Compared with the Pb group, the content of GSH/GSSG in BSP1+Pb, BSP3+Pb, and BSP4+Pb groups was enhanced 130%, 96%, and 118%, and the difference was statistically significant (*p* < 0.05). GSH/GSSG expression in the VC group also showed an improvement of 86% compared with Pb group (*p* < 0.05) (Figure 5F).

Glutathione is a vital reducing agent within cells, and its most important function is antioxidant. This study explored the effects of Pb and black soybean peptides on GPx, GR and GSH/GSSG in PC12 cells. The results showed that Pb exposure reduced the expression of GPx, GR, and GSH/GSSG. At the same time, three different black soybean peptides alleviated the cellular damage caused by Pb and restore the expression of GPx, GR, and GSH/GSSG to a certain extent. Wu et al. found that tert-butyl hydrogen peroxide (t-BHP) (100 μM) cotreated with PC12 cells for 1 h increased lipid ROS production, recovered Gps4 expression, and lowered the GSH/GSSG ratio [11]. Yi et al. found that the presence of H_2_O_2_ stimulated oxidative stress in HepG2 cells, characterized by the downregulation of GSH and a marked increase in GSSG levels [7].

### 3.5. The Influence of BSPs on Keap1/Nrf2/TXNIP Signal Pathway

Under normal physiological conditions, Nrf2, an essential transcription factor regulated by intracellular oxidative stress, and Keap1, a cytoplasmic chaperone protein molecule, bind to each other and maintain a relatively inhibited state [28]. The redox-sensitive repressor Keap1 mainly regulates of Nrf2 transcriptional activity. As a significant regulator in cells, Nrf2 fights oxidative stress by activating antioxidant stress proteins and phase II detoxification enzymes [34]. Keap1 includes a variety of stress sensors and inactivation modes that allow for a variety of cellular inputs, from oxidative stress and cellular metabolites to dysregulated autophagy, which can modulate Nrf2 activity [33]. The Keap1/Nrf2 system is integrated into several cell-signaling and metabolic pathways, making Nrf2 activation a crux regulatory node in some kinds of disease phenotypes [35]. The Nrf2/Keap1 axis has become a key regulator of intracellular homeostasis [14]. TXNIP has been studied to be an essential pathological regulator of disease, especially those associated with inflammation and glycolipid abnormalities. It works in conjunction with Keap1 and Nrf2 to relieve oxidative damage caused by the stimulation of cells [16].

**Figure 6:**
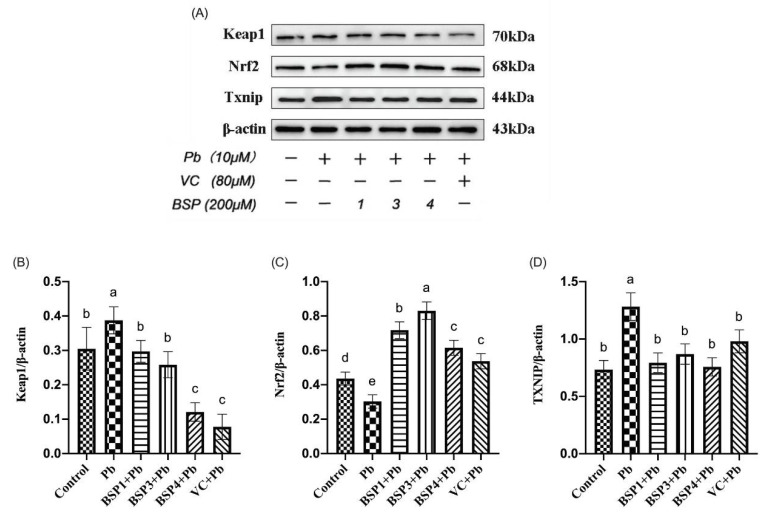
Related protein expression of Keap1/Nrf2/TXNIP signaling pathway. (**A**) WB results for Keap1, Nrf2 and TXNIP; (**B**) change in Keap1 protein expression relative to β-actin in different groups; (**C**) change in Nrf2 protein expression relative to β-actin in different groups; (**D**) change in TXNIP protein expression relative to β-actin in different groups. The value of the bars indicates the means ± SD (*n* = at least 3). The same letter means there is no significant difference, and different letters mean there is a significant difference (*p* < 0.05).

The Western blotting results of different treatments on Keap1, Nrf2, and TXNIP are shown in Figure 6A. As shown in Figure 6B, the Keap1 expression in the Pb group was increased by 27% compared with the control group. However, the three black soybean peptide + Pb groups were reduced by 30%, 43%, and 88% compared with the Pb group, and the difference was statistically significant. Keap1 expression in the VC group also showed an improvement of 102% compared with the Pb group (*p* < 0.05). Our study showed that BSP1, BSP3, and BSP4 alleviated lead-induced Keap1 upregulation in PC12 cells. As can be seen from Figure 6C, Nrf2 expression in the Pb group was decreased by 31% compared with the control group. However, the black soybean peptide + Pb groups were increased by 95%, 121%, and 71% compared with the Pb group (*p* < 0.05), and the difference was statistically significant (*p* < 0.05). Nrf2 expression in the VC group also showed an improvement of 54% compared with the Pb group (*p* < 0.05). Our study found that BSP1, BSP3, and BSP4 alleviated the downregulated expression of Nrf2 in lead-induced PC12 cells. As can be seen from Figure 6D, the expression of TXNIP in the Pb group was increased by 74% compared with the control group. In contrast, the expression of TXNIP in the BSP1+Pb, BSP3+Pb, and BSP4+Pb groups was decreased by 66%, 56%, and 69%, and the difference was statistically significant (*p* < 0.05). TXNIP expression in the VC group also showed an improvement of 41% compared with the Pb group (*p* < 0.05). Our study found that BSP alleviated the upregulation expression of TXNIP in lead-induced PC12 cells (*p* < 0.05).

The Pb group enhanced the expression of Keap1 and TXNIP while reducing the expression of Nrf2, but BSP1, BSP3 and BSP4 pretreatment groups decreased the expression of Keap1 and TXNIP while enhancing the expression of Nrf2, and the difference was statistically significant (*p* < 0.05).

It has been found that the peptide-containing group (PCG) produced by soy protein-derived peptides has a strong antioxidant effect on oxidative stress in H_2_O_2_-induced HepG2 cells [7]. PCG lowers levels of Keap1, which activates Nrf2 by reducing ubiquitination through the Keap1/Nrf2 pathway. Activated Nrf2 upregulates antioxidant enzyme activity (GSH-Px, CAT and SOD) and inhibits MDA and ROS production. It has been found that the miR-141 (microRNA-141)-mediated Keap1/Nrf2 signaling pathway promotes cell viability, reduces apoptosis, and decreases oxidative stress in PC12 cells, thus alleviating H/R (hypoxia/reoxygenation)-induced cell damage [27]. When the cell is stimulated, Nrf2 separates from Keap1. It enters the nucleus, which then binds to antioxidant response elements (AREs). Finally, it causes the expression of some antioxidant and metabolic genes, including TRX, which in turn reduces oxidative damage caused by various stimuli [36]. As an essential cellular antioxidant protein, TXNIP can fight oxidative stress and inhibit the apoptosis of cells. Nrf2 activity regulates oxidative stress in cells through the Keap1/Nrf2/TXNIP signaling pathway. Studies have shown that the piperine derivative HJ105 can alleviate human amyloid β peptide 1–42 (Aβ_1–42_)-induced cellular oxidative damage and neuroinflammation through the Keap1/Nrf2/TXNIP signaling pathway [37].

## 4. Conclusions

This study showed that Pb exposure significantly reduced PC12 cell viability, induced ROS and MDA production in PC12 cells, reduced SOD, CAT, GPx, GR, and GSH/GSSG expression, and increased Keap1, Nrf2, and TXNIP expression. However, these three black soybean peptides can alleviate these phenomena induced by Pb. These three kinds of black soybean peptides (BSP1, BSP3, and BSP4) can alleviate lead-induced oxidative stress damage in PC12 cells, and this process may be through the Keap1/Nrf2/TXNIP signaling pathway.

## Figures and Tables

**Table 1:** The static charge, biological activity, toxicity, and water solubility of BSPs.

NO.	Sequence	Static Charge	Activity Score	Toxicology	Water-Solubility
BSP1	KKWNP	+2	0.498	Atoxic	Good
BSP2	KKAFPKD	+2	0.289	Atoxic	Good
BSP3	KAKSPLF	+2	0.688	Atoxic	Good
BSP4	KKATNPLF	+2	0.579	Atoxic	Good
BSP5	KKKILSYAMDG	+2	0.227	Atoxic	Good

## Abbreviations

Pb	Lead
BSP	Black soybean peptide
CCK8	Cell Counting Kit 8
SOD	Superoxide dismutase
CAT	Catalase
MDA	Malondialdehyde
GR	Glutathione reductase
GPx	Glutathione peroxidase
ROS	Reactive oxygen species
GSH	Glutathione
GSSG	Oxidized glutathione
Nrf2	Nuclear Factor erythroid 2-related factor 2
Keap1	Kelch-like ECH-associated protein 1
TXNIP	Thioredoxin-interacting protein
TRX	Thioredoxin
